# Conditional Loss of *Hoxa5* Function Early after Birth Impacts on Expression of Genes with Synaptic Function

**DOI:** 10.3389/fnmol.2017.00369

**Published:** 2017-11-15

**Authors:** Benoit Lizen, Charlotte Moens, Jinane Mouheiche, Thomas Sacré, Marie-Thérèse Ahn, Lucie Jeannotte, Ahmad Salti, Françoise Gofflot

**Affiliations:** ^1^Institut des Sciences de la Vie, Université catholique de Louvain, Louvain-la-Neuve, Belgium; ^2^Department of Molecular Biology, Medical Biochemistry and Pathology, Université Laval, Quebec City, QC, Canada; ^3^Centre de Recherche sur le Cancer, Université Laval, Quebec City, QC, Canada; ^4^Centre de Recherche, Centre Hospitalier Universitaire de Québec, Université Laval, Quebec City, QC, Canada

**Keywords:** RNA sequencing, conditional mutagenesis, calcium signaling, glutamatergic synapse, GABAergic synapse, brainstem

## Abstract

*Hoxa5* is a member of the *Hox* gene family that plays critical roles in successive steps of the central nervous system formation during embryonic and fetal development. In the mouse, *Hoxa5* was recently shown to be expressed in the medulla oblongata and the pons from fetal stages to adulthood. In these territories, *Hoxa5* transcripts are enriched in many precerebellar neurons and several nuclei involved in autonomic functions, while the HOXA5 protein is detected mainly in glutamatergic and GABAergic neurons. However, whether HOXA5 is functionally required in these neurons after birth remains unknown. As a first approach to tackle this question, we aimed at determining the molecular programs downstream of the HOXA5 transcription factor in the context of the postnatal brainstem. A comparative transcriptomic analysis was performed in combination with gene expression localization, using a conditional postnatal *Hoxa5* loss-of-function mouse model. After inactivation of *Hoxa5* at postnatal days (P)1–P4, we established the transcriptome of the brainstem from P21 *Hoxa5* conditional mutants using RNA-Seq analysis. One major finding was the downregulation of several genes associated with synaptic function in *Hoxa5* mutant specimens including different actors involved in glutamatergic synapse, calcium signaling pathway, and GABAergic synapse. Data were confirmed and extended by reverse transcription quantitative polymerase chain reaction analysis, and the expression of several HOXA5 candidate targets was shown to co-localize with *Hoxa5* transcripts in precerebellar nuclei. Together, these new results revealed that HOXA5, through the regulation of key actors of the glutamatergic/GABAergic synapses and calcium signaling, might be involved in synaptogenesis, synaptic transmission, and synaptic plasticity of the cortico-ponto-cerebellar circuitry in the postnatal brainstem.

## Introduction

The HOX family of transcription factors gathers key regulators of embryo patterning, organ development, and cell differentiation during animal development, but also throughout adult life (Mallo et al., 2010; Rezsohazy et al., 2015). In the central nervous system (CNS) *Hox* genes play critical roles in successive developmental steps, from neurulation to the establishment of neuronal networks (Nolte and Krumlauf, 2007; Dasen and Jessell, 2009; Narita and Rijli, 2009; Di Bonito et al., 2013a; Karmakar et al., 2017). During neurulation, combinatorial expression of *Hox* genes provides a segmental identity and antero-posterior patterning information to neural progenitors (Nolte and Krumlauf, 2007). Some *Hox* genes remain expressed in the CNS after neurulation and they are implicated in normal tangential migration of pontine neurons, in axonal connection and arborization, in topographic connectivity, in synaptic refinement and in neuron survival (Oury et al., 2006; Pasqualetti et al., 2007; Geisen et al., 2008; Di Bonito et al., 2013b; Philippidou and Dasen, 2013; Bechara et al., 2015; Catela et al., 2016; Karmakar et al., 2017). Finally, recent evidences show that *Hox* genes from paralog groups 3–8 (PG3–8) display differential anterior limits of expression in the perinatal and adult hindbrain derivatives suggesting a continuing role in differentiation of neuronal derivatives after birth (Hutlet et al., 2016; Tomas-Roca et al., 2016).

*Hoxa5*, a member of *Hox* PG5, is known for its numerous activities during development (Jeannotte et al., 2016) and, together with its paralog genes, for its implication in different processes of fetal CNS development (Philippidou et al., 2012; Di Meglio et al., 2013; Catela et al., 2016). In the hindbrain at fetal stages, *Hoxa5* expression is detected in the caudal part of the medulla oblongata and in the posterior pontine region, where it participates in the tangential migration of precerebellar neurons from the rhombic lip toward the pontine gray (PG) nucleus (Di Meglio et al., 2013; Tomas-Roca et al., 2016; Lizen et al., 2017). Indeed, PG5 HOX proteins are shown to be functionally implicated in the topographic organization of migrating precerebellar pontine neurons, through the negative regulation of *Unc5b* expression, a netrin repulsive receptor (Di Meglio et al., 2013). In the spinal cord, lack of *Hoxa5* and *Hoxc5* gene functions in mutant mice results in an important reduction and disorganization of motor neurons from the phrenic motor column, severe defects in diaphragm innervation, and death at birth (Philippidou et al., 2012). PG5 genes also contribute with other *Hox* genes to neural fate, organization, and peripheral connectivity of spinal cord motor neurons targeting the forelimb, through regulation of *Ret* and *Gfra* genes (Catela et al., 2016). Recently, our detailed analysis of *Hoxa5* expression pattern in the mouse brain revealed that *Hoxa5* is still expressed after birth until adulthood. A key feature of this analysis was the enrichment of *Hoxa5* transcripts in pontine and medullary precerebellar neurons. They are also detected in different nuclei implicated in the control of autonomic functions (Lizen et al., 2017). In these territories, the HOXA5 protein is specifically present in neurons, and principally in glutamatergic and GABAergic neurons. This mapping analysis led us to hypothesize *Hoxa5* functions in CNS at fetal and postnatal stages, such as having a potential role in the establishment, refinement, or plasticity of precerebellar circuits during postnatal and adult life. To test these hypotheses and to provide insight into the HOXA5 functions in the brain after birth, we aimed in the present paper at identifying the transcriptional programs and main biological processes downstream of HOXA5 in the postnatal brainstem.

To achieve this objective, we performed a RNA-Seq transcriptomic analysis using a mouse model carrying a conditional postnatal loss-of-*Hoxa5* function mutation. Using this approach, we bypassed the early requirement for HOXA5 in the fetal hindbrain and directly tackled HOXA5 functions at the postnatal phase of brainstem development. This paradigm allowed to identify genes that are differentially expressed in *Hoxa5*-inactivated brainstem when compared with *Hoxa5*-expressing brainstem. We expect that understanding of the transcriptional programs downstream of HOXA5 will serve as a stepstone for further functional analysis of this transcription factor in the postnatal hindbrain at a critical period for remodeling and maturation of neuronal circuits.

## Materials and Methods

### Animals

Mice were maintained in a conventional facility and fed in standard conditions (mice maintenance and mice breeding diets, Carfil Quality, Belgium) on a 14 h light/10 h dark cycle. Experimental procedures on animals were performed in accordance with the Belgian national guidelines and in agreement with the European directive 2010/63/UE. The protocol was approved by the animal ethic committee of the Université catholique de Louvain (approval 122803). For all gas euthanasia, CO_2_ was administered by progressive delivery in the cage volume in accordance with guidelines. The transgenic *CMV-CreER*^T2^ mice and the characterization of Cre activity with reporter mice have been described elsewhere (Santagati et al., 2005; Lizen et al., 2015). We used the *Hoxa5*^tm1.1Ljea^ mouse line carrying a *Hoxa5* floxed allele as described (Tabaries et al., 2007). The characterization of Cre efficiency on the *Hoxa5* floxed allele after tamoxifen induction has already been described (Lizen et al., 2015). Genotyping of animals was accomplished by polymerase chain reaction (PCR) using the primers and the PCR program described in Supplementary Table S1.

*CMV-CreER*^T2^ transgenic mice were bred with mice carrying the conditional *Hoxa5* floxed allele to generate double transgenic animals homozygous for the *Hoxa5* floxed allele (*Hoxa5*^flox/flox^) and heterozygous for the *CMV-CreER*^T2^ transgene. Compound transgenic *Hoxa5*^flox/flox^*;CMV-CreER*^T2+/-^ males were bred with *Hoxa5*^flox/flox^ females. These crosses had the advantage of producing in the same litter pups that were either CreER^T2^ positive or CreER^T2^ negative, providing intra-litter controls. The *Hoxa5*^flox/flox^*;CMV-CreER*^T2+/-^ mice were used in a mixed background composed principally of 129/Sv, with a minor contribution of C57BL/6 and DBA/2.

### Tamoxifen Administration

For induction treatments, tamoxifen (Sigma–Aldrich, T5648) was dissolved in corn oil (Sigma–Aldrich, C8267). Intragastric injections of 50 μl of three different stock solutions were performed: we injected the first solution of 1 mg/ml at postnatal day (P)1, the second of 1.5 mg/ml was injected at P2 and P3, and the last one of 2 mg/ml was injected at P4 (Lizen et al., 2015). Pups stayed with their mother during the whole treatment until they were euthanized at P21. For euthanasia, CO_2_ was administered by progressive delivery in the cage volume in accordance with guidelines.

### Sample Preparation

Brains were collected after euthanasia as described above. For RNA-Seq experiments, whole brainstems were collected while for reverse transcription quantitative PCR (RT-qPCR) assays, the pons and caudal part of the medulla oblongata were dissected as illustrated in Supplementary Figure S1. Tissues were then snap frozen in liquid nitrogen, and stored at -80°C until RNA was extracted. RNA was isolated using TRI Reagent (Sigma–Aldrich, T9424) and quantified using a NanoDrop ND-1000 fluorospectrometer (Thermo Fisher Scientific, Waltham, MA, United States).

### RNA-Seq Analyses

RNA sequencing analysis was carried out on total RNA extracted from tamoxifen-treated individuals of two different genotypes: *Hoxa5*^flox/flox^*;CMV-CreERT*^2+/-^ (*Hoxa5* cKO) and *Hoxa5*^flox/flox^*;CMV-CreERT*^2-/-^ (*Hoxa5* control). To minimize sample variability caused by individual differences between animals linked to tamoxifen treatment, RNA from three brainstems was pooled, and three pools were used per genotype (for a total of nine samples per genotype) (Supplementary Figure S1). Library preparation and sequencing were performed at the Genomics Facility of the Interdisciplinary Cluster for Applied Genoproteomics (GIGA Genomics, ULg-Liège, Belgium). RNA integrity was verified on the Bioanalyzer 2100 with RNA 6000 Nano chips, RNA integrity number scores were more than 8 for all samples. Illumina truSeq stranded total RNA library prep kit with ribo-zero was used to prepare libraries from 1 μg of total RNA following Illumina’s protocol. Ribosomal RNA was depleted from total RNA. The remaining RNA was purified, fragmented, and then primed with random hexamers and reverse transcribed into first strand cDNA followed by second strand synthesis. A single A nucleotide was added to the 3′end of the fragments before ligation to multiple indexing adapters. DNA fragments with adapter molecules on both ends were enriched by PCR and purified with Ampure XP magnetic beads. Libraries were validated on Bioanalyzer DNA 1000 chip and quantified by qPCR with the KAPA library quantification kit. Sequencing was performed on Illumina NextSeq 500 with NextSeq 500 High Output V2 kit (2 × 75 cycles). For data analysis, Fastq files were trimmed for adaptor sequences. The reads were aligned with Tophat 2.0.9^1^ to the mouse genome. Cufflinks 2.2.0 suite^2^ was used to generate fragments per kilobase of transcript per million fragments mapped values. CuffDiff was also used to identify significantly differentially expressed genes between the two genotypes. Statistical significance was defined by the Benjamini–Hochberg method. The *q*-value is a corrected *p*-value. The *q*-values below 0.05 were considered as a significant difference of gene expression between the two conditions. Gene ontology (GO) term analysis was performed using DAVID 6.7 Bioinformatics resource tools (Huang da et al., 2009). High-throughput sequencing data used in this publication have been deposited in NCBI’s Gene Expression Omnibus (Edgar et al., 2002) and are accessible through GEO Series accession number GSE103390^3^.

### RT-qPCR Analyses

Brain subregions were homogenized in 1 ml of TRI Reagent (Sigma–Aldrich, T9424) during 30 s using high-throughput tissue homogenizer (Precellys^®^24). RNA was isolated from the dissected brain subregions following manufacturer’s instructions. The precipitated RNA pellet was dissolved in 100 μl of nuclease free water and reverse-transcription was performed using a reverse transcription kit (Qiagen, 205311) according to the manufacturer’s instructions. Hoxa5 and target genes expression was assessed by qPCR on a StepOne+ apparatus (Applied Biosystems) using SYBR Green (Qiagen, 204143) as the detection method, and the geometric mean of two reference genes (H2A and Gapdh) as internal normalization reference. The relative transcripts abundance (fold change) was calculated using the 2^-ΔΔC_T_^ calculation, where ΔCt = Ct_genetarget_ - Ct_referencegenes_ and ΔΔCt = ΔCt_mutant_ - ΔCt_control_. **Supplementary Table S2** provides primers sequences and concentrations, as well as the efficiency of the amplification reaction.

### Non-radioactive *in Situ* Hybridization

Eight sets of 14 μm thick serial coronal cryosections per brain were cut on a Leica CM 3050S cryostat. Gene expression was detected using digoxigenin-labeled RNA probes, as previously described, and as optimized for *Hox* genes analysis in the adult brain (Chotteau-Lelievre et al., 2006; Hutlet et al., 2016).

Plasmids allowing *in vitro* transcription of digoxigenin-labeled riboprobes were obtained from collaborators (*En2*: Dr A. Joyner; *NeuroD2*: M. Studer; *Wnt7a*: N. Bobbola). For other genes, probes were transcribed directly from PCR amplification products, following an adaptation of the Allen Brain Atlas procedures. For probes synthesis, cDNA of adult mouse brain was used to target genes of interest by a first PCR with designed gene-specific primers (NCBI primer blast). For practical purposes, the T7 universal primer sequence (5′-GCGTAATACGACTCACTATAGGG-3′) was directly attached to the forward and the Sp6 one (5′-ATTTAGGTGACACTATAG-3′) to the reverse primer. PCR products were then amplified by a second PCR reaction, purified using a PCR purification kit (Qiagen, 28104), and eluted with 30 μl of elution buffer (10 mM Tris, pH 8.5). *In vitro* transcription with SP6 and T7 polymerases was performed on the resulting eluate to generate both RNA sense and anti-sense probes. Probes size and quality were evaluated following *in vitro* transcription by migration on agarose gel. Prior use for *in situ* hybridization (ISH), all probes were further diluted to a concentration of 100 ng/μl of hybridization mix (Life Technologies, B8807G), and stored at -20°C. A detailed list of data regarding specific probe primers and sequences is available upon request.

Double ISH was performed on cryostat sections using a combination of digoxigenin- and fluorescein-labeled probes as described (Mayer et al., 2010). Anti-Fluorescein (Roche, 11426338910, 1:3000) immunodetection was used first to reveal candidate target genes, in combination with NBT-BCIP for the first staining reaction. The reaction was stopped with PBT/EDTA. Sections were washed 2 × 10 min in maleic acid buffer with 0.1% Tween 20 (MABT) (Sigma–Aldrich, 9005-64-5). Sections were then incubated in a blocking solution containing MABT with 2% of blocking reagent (Roche, 11 096 176 001) and 20% heat-inactivated goat serum (Sigma–Aldrich, G6767). To target the *Hoxa5* probe, the sections were then incubated with anti-digoxigenin antibody (Roche, 11093274910, 1/1500) and INT-BCIP (Roche, 11 681 460 001) were used for the second staining reaction.

Hybridized sections were observed on a Leica DM2500 microscope, and pictures were captured with a Leica DFC420C camera. Annotation of expression thresholds versus background were established for each ISH taking into account the appropriate control and the duration of the staining reaction. A frequently used control for specific hybridization to mRNA is a sense strand probe which should not specifically anneal to the target RNA. In this study, we used as a negative control an antisense probe for a *Hox* gene not expressed in the brain (e.g., Hoxa10) and an antisense probe for *Gad67* as a positive control.

## Results

### HOXA5 Regulates Multiple Genes Involved in Synaptic Function and Calcium Pathway in Postnatal Brainstem

**FIGURE 1:**
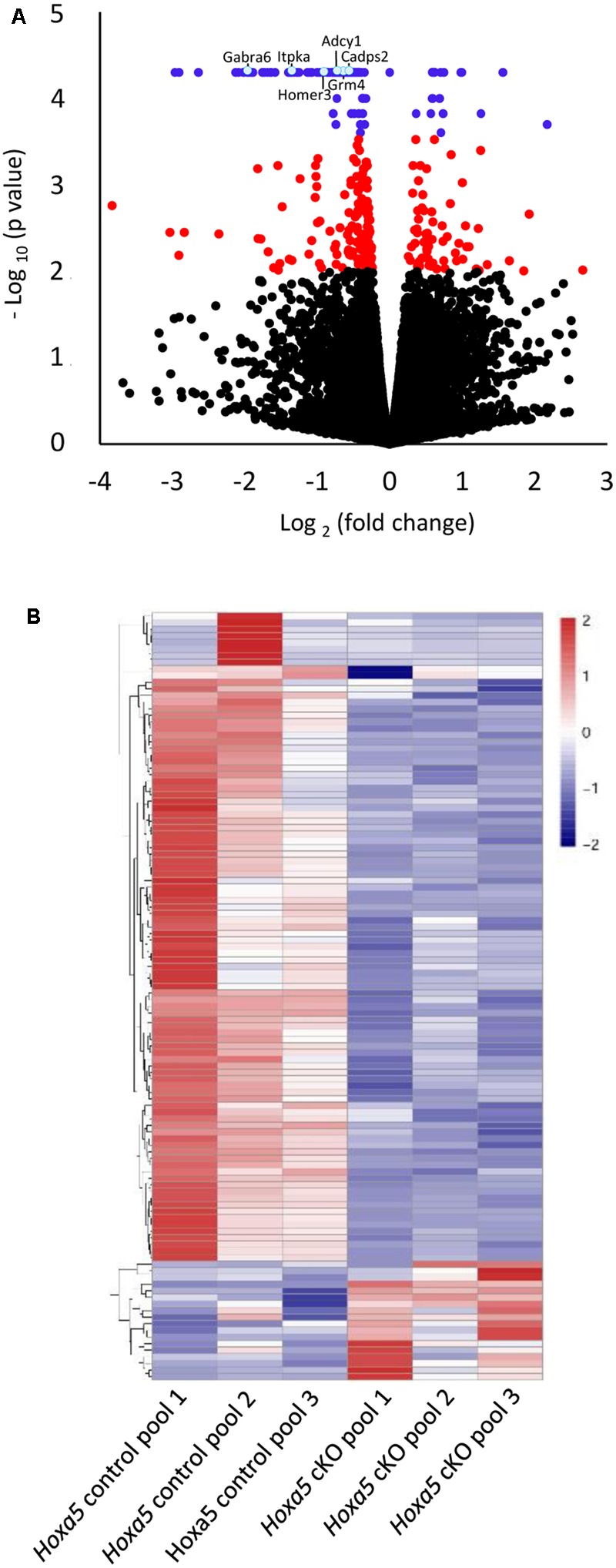
Transcriptome analysis of P21 brainstem in tamoxifen-treated *Hoxa5*^flox/flox^*;CMV-CreER*^T2-/-^ (*Hoxa5* control) and tamoxifen-treated *Hoxa5*^flox/flox^*;CMV-CreER*^T2+/-^ (*Hoxa5* cKO) (*n* = 3 pools of 3 individuals per genotype). **(A)** Volcano plot (*p*-value versus fold change) of differentially expressed transcripts. Transcripts whose abundance level is significantly modified in *Hoxa5* cKO (*q*-value <0.05) are colored in blue, while those with a *q*-value >0.05 and a *p*-value <0.01 are colored in red. **(B)** Heatmap showing hierarchical clustering of significantly downregulated (blue) and upregulated (red) transcripts (*q*-value <0.05) in the *Hoxa5* control pools and in the *Hoxa5* cKO pools. A large proportion of significantly downregulated transcripts are detected in the *Hoxa5* cKO pools. See Supplementary Table S3 for complete list of genes.

To explore the HOXA5 transcriptional landscape in the postnatal brainstem, we carried out transcriptomic analyses by RNA-Seq using a model of postnatal *Hoxa5* loss-of-function. We induced *Hoxa5* inactivation after birth (P1–P4) using the tamoxifen-inducible CMV-CreER^T2^ mice and conditional *Hoxa5* floxed allele mice (*Hoxa5*^flox^) (Santagati et al., 2005; Tabaries et al., 2007; Lizen et al., 2015). This treatment results in a severe reduction of *Hoxa5* transcripts in brainstem subregions (8–12%, see below, RT-qPCR analysis) and of HOXA5-positive cells in the brain (7–26% depending on nuclei) (Lizen et al., 2015). RNA was extracted from the brainstem of P21 tamoxifen-treated *Hoxa5*^flox/flox^*;CMV-CreER*^T2+/-^ (herein called *Hoxa5* cKO) pups and from tamoxifen-treated *Hoxa5*^flox/flox^*;CMV-CreER*^T2-/-^ (herein called *Hoxa5* control) littermates. To identify differentially expressed genes, we performed three independent replicate experiments, in which each replicate contains RNA pooled from three brainstems (see section “Materials and Methods”; Supplementary Figure S1). Transcriptome analysis revealed 116 transcripts whose expression levels were significantly modified in brainstem from P21 mutants (*q*-value <0.05) and 318 transcripts with a *p*-value lower than 0.01 (**Figure 1A** and Supplementary Table S3). Among the 116 transcripts, 98 (84.5%) were downregulated and 18 (15.5%) were upregulated in mutants (**Figure 1** and Supplementary Table S3). The relative abundance levels of transcripts that are significantly modified are illustrated for each of the three pooled samples in the heat map (**Figure 1B**).

**FIGURE 2:**
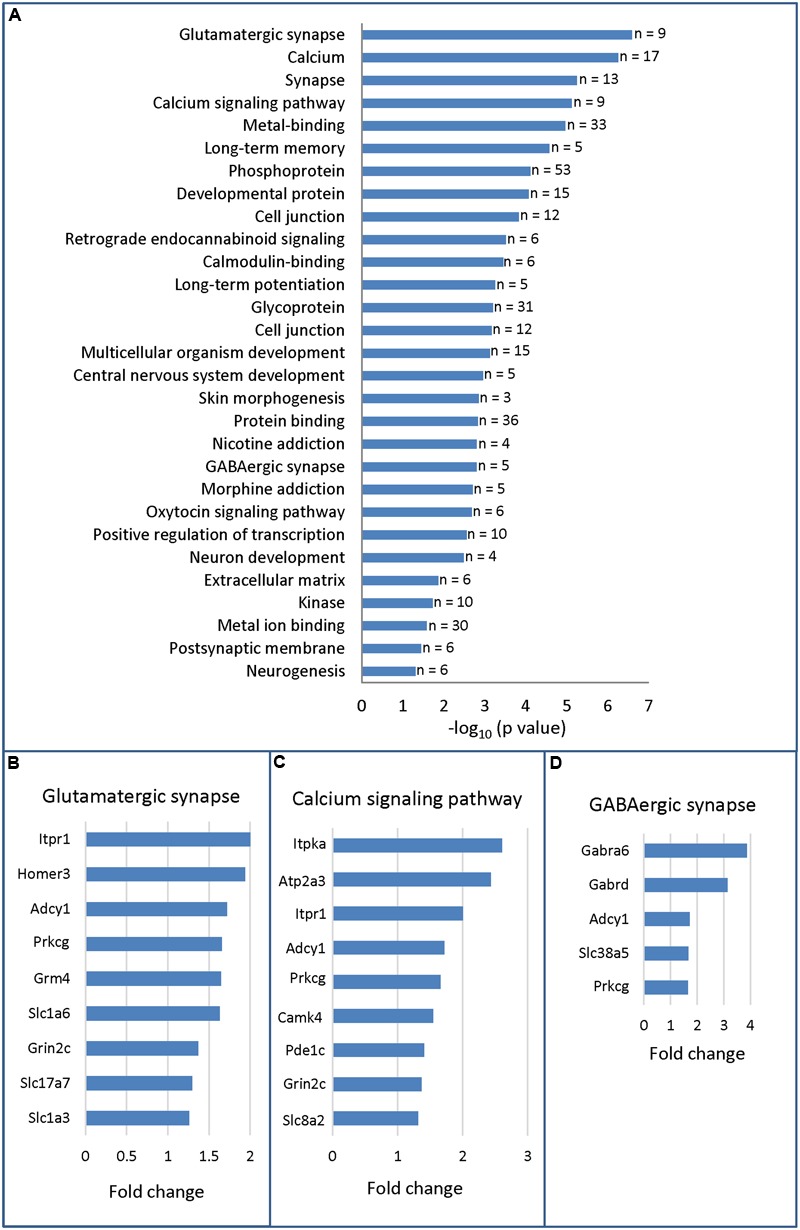
Gene ontology analysis (DAVID 6.7) of RNA-sequencing data from P21 brainstem in *Hoxa5* control and *Hoxa5* cKO. **(A)** Functional annotation chart. Significantly enriched terms (*p* < 0.05) associated to differentially expressed transcripts are presented in descending order (–log_10_ of *p*-value in *x*-axis). As detailed in Supplementary Table S4, *p*-values of the significantly enriched terms vary between 2.6 × 10^-7^ and 4.6 × 10^-3^. The number of genes for each term is indicated on the right. **(B)** Genes identified in the glutamatergic synapse term. **(C)** Genes identified in the calcium signaling pathway term. **(D)** Genes identified in the GABAergic synapse term. Fold change (*x*-axis) in **(B–D)** is the ratio between transcript abundance value measured in *Hoxa5* control and transcript abundance value measured in *Hoxa5* cKO in RNA-Seq analysis (see Supplementary Table S3 for detailed values).

To gain insights into the molecular programs regulated by HOXA5, we applied a GO analysis on the 116 differentially expressed genes. The top 3 most significant enriched terms were respectively glutamatergic synapse (*p*-value = 2.6 × 10^-7^), calcium (*p*-value = 5.6 × 10^-7^), and synapse (*p*-value = 5.7 × 10^-6^), and included only downregulated genes (**Figure 2A** and Supplementary Table S4). A striking feature of this analysis is the importance of enriched terms associated with synapse such as glutamatergic synapse, calcium, synapse, calcium signaling pathway, long-term memory, cell junction, GABAergic synapse, long-term potentiation, postsynaptic membrane (**Figure 2A**). Further analyses through NCBI^4^ allowed to identify a total of 39 genes with evidence of synaptic function among the 98 downregulated genes (∼40%). In contrast, we did not identify any genes with evidence of function at the synapse among the upregulated transcripts in mutant brains.

Detailed analysis of candidate target genes involved in glutamatergic synapse revealed downregulation of several receptors and transporters, such as the metabotropic glutamate receptor *Grm4*, the subunit of the *N*-methyl-D-aspartate (NMDA) receptor *Grin2c*, the L-glutamate transmembrane transporters *Slc1a6 (Eaat4)* and *Slc1a3 (Eaat1, Glast)* and the vesicular glutamate transporter *Slc17a7 (Vglut1)* (**Figure 2B**). Downstream effectors, such as *Prkcg*, the member of the PKC family that regulates NMDA receptors trafficking (Lan et al., 2001), and the synaptic plasticity modulator *Adcy1* were also downregulated (Wang and Zhang, 2012). Other candidate targets include members of the inositol 1,4,5-trisphosphate (IP3) pathway: *Itpr1* (inositol 1,4,5-trisphosphate receptor), which mediates calcium release from the endoplasmic reticulum, and its interactor *Homer3* (**Figure 2B**) (Kammermeier, 2006; Mizutani et al., 2008; Goto and Mikoshiba, 2011). Several of these targets are also influenced by calcium or involved in calcium signaling: *Itpr1, Adcy1, Prkcg*, and *Grin2c* (**Figure 2C**) (Oancea and Meyer, 1998; Wong et al., 1999; Goto and Mikoshiba, 2011). Additional downregulated targets involved in calcium signaling pathway are: *Camk4*, the calcium/calmodulin-dependent protein kinase IV involved in neural calcium signaling; *Atp2a3*, the ATPase endoplasmic reticulum Ca^2+^ transporting 3; *Itpka*, the inositol-trisphosphate 3-kinase; and *Car8*, the carbonic anhydrase 8 which regulates calcium homeostasis through ITPR1 inhibition (Zhuang et al., 2015). Finally, downregulated transcripts involved in GABAergic synapse included the two GABA receptor subunits *Gabra6* and *Gabrd*, which were highly downregulated in mutants (fold change >3) and the System N Glutamine Transporter *Slc38a5* (SNAT5) (**Figure 2D**). As indicated by the two enriched GO terms postsynaptic membrane and postsynaptic cell membrane (**Figure 2A** and Supplementary Table S4), most of the downregulated transcripts involved in glutamatergic synapse and GABAergic signal are mainly postsynaptic.

In summary, deletion of *Hoxa5* gene function early after birth causes downregulation of numerous genes involved in synaptic function in the brain few weeks after. Notably, transcripts of multiple genes coding major players of glutamate and GABA neurotransmission, as well as key actors of calcium signaling, were downregulated. Due to the absence of highly enriched terms associated to upregulated genes (Supplementary Table S5), further analysis was carried out only for downregulated genes.

### RT-qPCR Analysis of HOXA5 Candidate Targets in the Pons and Medulla Oblongata

A subset of candidate HOXA5 target genes identified by RNA-Seq analysis was analyzed by real-time RT-qPCR. Selection of genes was based on GO functional annotations, expression fold changes, and literature. Genes that were downregulated at least 1.5-fold in *Hoxa5* cKO brainstem (Supplementary Table S4) and known to be functionally related to synapse and calcium signaling (**Figures 2B–D**) were favored. *Zic* genes encoding transcription factors were also selected based on their known expression and critical function in mossy fibers precerebellar neurons (Dipietrantonio and Dymecki, 2009). For quantitative analysis through RT-qPCR, we improved the resolution of the analysis by measuring RNA levels of the selected genes in dissected pons and caudal part of the medulla oblongata since HOXA5 expression was shown to be enriched in these restricted areas (Supplementary Figure S1) (Lizen et al., 2017).

**FIGURE 3:**
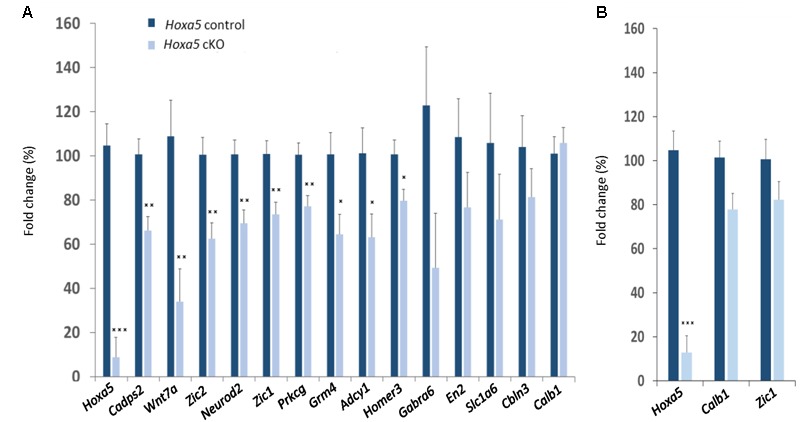
RT-qPCR analysis of selected candidate target genes of HOXA5 in the medulla oblongata **(A)** and pons **(B)** of tamoxifen-treated *Hoxa5*^flox/flox^*;CMV-CreER*^T2-/-^ (*Hoxa5* control; dark blue) and tamoxifen-treated *Hoxa5*^flox/flox^*;CMV-CreER*^T2+/-^ (*Hoxa5* cKO; light blue) at P21. Data are reported as means ± SEM. Asterisks depict statistically significant differences compared with *Hoxa5* control brains: ^∗∗∗^*p* < 0.001, ^∗∗^*p* < 0.01, and ^∗^*p* < 0.05. *p*-Values are from ANOVA test and are provided in **Table 1**. *n* = 3–6 for *Hoxa5* control brains, *n* = 3–8 for *Hoxa5* cKO brains. The relative transcripts abundance is calculated using the 2^-ΔΔCt^ method as detailed in Section “Materials and Methods,” where normalized values measured in *Hoxa5* cKO are reported to normalized values measured in *Hoxa5* control. The fold change is presented as a percentage of this value: (2^-ΔΔCt^) × 100.

RT-qPCR confirmed downregulation of *Cadps2, Wnt7a, Zic2, Neurod2, Zic1, Prkcg, Grm4, Adcy1*, and *Homer3* in the dissected medulla oblongata of *Hoxa5* cKO specimens (*p*-value <0.05; **Table 1**). For *Gabra6, Cbln3, En2*, and *Slc1a6*, transcript levels were reduced in *Hoxa5* cKO samples compared to *Hoxa5* control but did not reach the significance (*p*-value between 0.1 and 0.3) (**Figure 3A** and **Table 1**). In the pons of *Hoxa5* cKO, *Calb1* was the only candidate for which the downregulation was close to the level of significance (*p*-value 0.0519), but the level of *Zic1* was also reduced in *Hoxa5* cKO samples compared to *Hoxa5* control, although it did not reach the significance (*p*-value 0.169) (**Figure 3B** and **Table 1**). Indeed, although we observed reduced transcription levels for several other candidate genes, the variability between the samples, possibly explained by the absence of precise landmarks for macro-dissection, prevented us from concluding.

**Table 1:** RT-qPCR analysis of selected candidate target genes of HOXA5 in the medulla oblongata and pons.

	Target genes	Fold change	*p*-value
Medulla	*Cadps2*	1.52	0.0052^∗∗^
Oblongata	*Wnt7a*	3.21	0.0076^∗∗^
	*Zic2*	1.61	0.0061^∗∗^
	*NeuroD2*	1.45	0.0063^∗∗^
	*Zic1*	1.37	0.0088^∗∗^
	*Prkcg*	1.30	0.0091^∗∗^
	*Grm4*	1.56	0.0243^∗^
	*Adcy1*	1.60	0.0381^∗^
	*Homer3*	1.26	0.0463^∗^
	*Gabra6*	2.49	0.0672
	*En2*	1.42	0.2089
	*Slc1a6*	1.49	0.2856
	*Cbln3*	1.27	0.2662
	*Calb1*	0.95	0.6538
Pons	*Calb1*	1.30	0.0519
	*Zic1*	1.22	0.1649

*p-values are from ANOVA test. *n* = 3–6 for *Hoxa5* control brains, *n* = 3–8 for *Hoxa5* cKO brains. Asterisks depict statistically significant differences in *Hoxa5* cKO brains compared with *Hoxa5* control brains: ^∗∗^*p* < 0.01 and ^∗^*p* < 0.05.*

Thus, RT-qPCR data confirmed the RNA-Seq results, thereby validating the robustness of the analysis. Moreover, this analysis suggested region-specific regulation of HOXA5 downstream genes. This prompted us to evaluate the neuroanatomical expression profiles of HOXA5 candidate target genes.

### Anatomical and Cellular Localization of HOXA5 Candidate Targets Expression in the Postnatal Brainstem

To gain insights into the expression pattern of candidate target genes in brainstem, ISH was performed on the brain of wild-type mice at P21. Probes were designed for the subset of target genes analyzed by RT-qPCR (see above for criteria). Reliable labeling was detected for *Calb1, Cadps2, Camk4, Cbln1, Grin2c, Itpr1, Neurod2, Vglut1, Wnt7a, Zic1*, and *Zic2* in *Hoxa5*-expressing nuclei (**Table 2**).

**Table 2:** Neuroanatomical localization of HOXA5 candidate target genes expression in *Hoxa5*-expressing nuclei, as evaluated by *in situ* hybridization analysis.

	HOXA5 positive nuclei	Detected transcripts
Medulla	XII (hypoglossal nucleus)	*Calb1, Itpr1, Vglut1, Wnt7a*
Oblongata	X (nucleus x)	*Cbln1, Grin2c, Vglut1, Zic1*
	SPIV (spinal vestibular nucleus)	*Cadps2*
	MV (medial vestibular nucleus)	*Cadps2, Grin2c, Zic1*
	PRP (nucleus prepositus)	*Cadps2, Grin2c, Zic1*
	PARN (parvicellular reticular nucleus)	*Calb1, Cbln1*
	MDRN (medullary reticular nucleus)	*Calb1*
	IRN (intermediate reticular nucleus)	*Itpr1*
	GRN (gigantocellular reticular nucleus)	*Cadps2, Cbln1*
	DMX (dorsal motor nucleus of the vagus nerve)	*Cadps2, Calb1*
	LRN (lateral reticular nucleus)	*Camk4, Cbln1, NeuroD2, Vglut1, Zic1, Zic2*
	SPVI (spinal nucleus of the trigeminal, interpolar part)	*Cbln1, Itpr1, Vglut1*
	SPVC (spinal nucleus of the trigeminal, caudal part)	*Adcy1, Calb1, Cbln1*
	NTS (nucleus of the solitary tract)	*Calb1, Cbln1, Zic1*
	ECU (external cuneate nucleus)	*Camk4, Cbln1, Grin2c, Vglut1, Zic1, Zic2*
	GR (gracile nucleus)	*Cbln1*
	CU (cuneate nucleus)	*Cbln1*
	AP (area postrema)	*Zic1*
Pons	IF5 (interfascicular trigeminal nucleus)	*Cbln1, NeuroD2, Vglut1, Zic1*
	TRN (tegmental reticular nucleus)	*Camk4, NeuroD2, Vglut1, Zic1, Zic2*
	PG (pontine gray)	*Camk4, NeuroD2, Vglut1, Zic1, Zic2*
	KF (koelliker-fuse subnucleus)	*Calb1*

**FIGURE 4:**
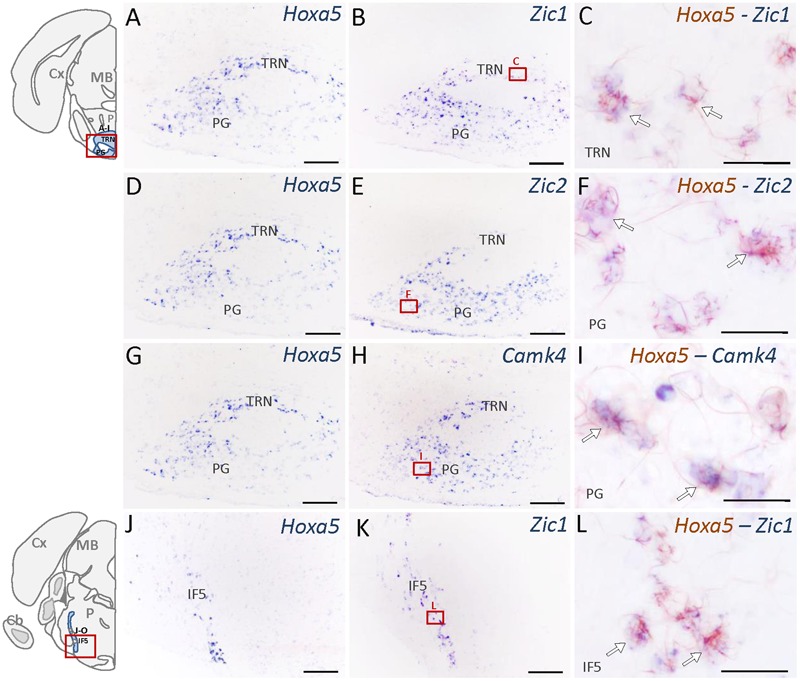
Expression of HOXA5 candidate target genes in the pons analyzed by single and double *in situ* hybridization (ISH) on coronal cryosections of P21 mouse brains. Schematic views of coronal brain sections illustrate brain structures while square red boxes on the schemes refer to the localization of single ISH pictures. In double ISH (right column) *Hoxa5* signal is in red/brown precipitate while target gene signal is in blue. Localization of double ISH pictures is illustrated by square red boxes on the single ISH pictures (middle column). **(A–C)** Signal of *Hoxa5* and *Zic1* in the pontine gray (PG) and tegmental reticular nucleus (TRN). **(D–F)** Signal of *Hoxa5* and *Zic2* in PG and TRN. **(G–I)** Signal of *Hoxa5* and *Camk4* in PG and TRN. **(J–L)** Signal of *Hoxa5* and Zic1 in the interfascicular trigeminal nucleus (IF5). As illustrated in the right column, many *Hoxa5*-positive cells also show labeling for target gene transcripts (white arrows). *n* = 2–4 brains hybridized for *Hoxa5* and each target genes in single and double ISH. Cb, cerebellum; Cx, cortex; MB, mid brain; P, pons. Medial is on the right, lateral is on the left. Scale bar 200 μm in **(A,B,D,E,G,H,J,K)**; 100 μm in **(C,F,I,L)**.

**FIGURE 5:**
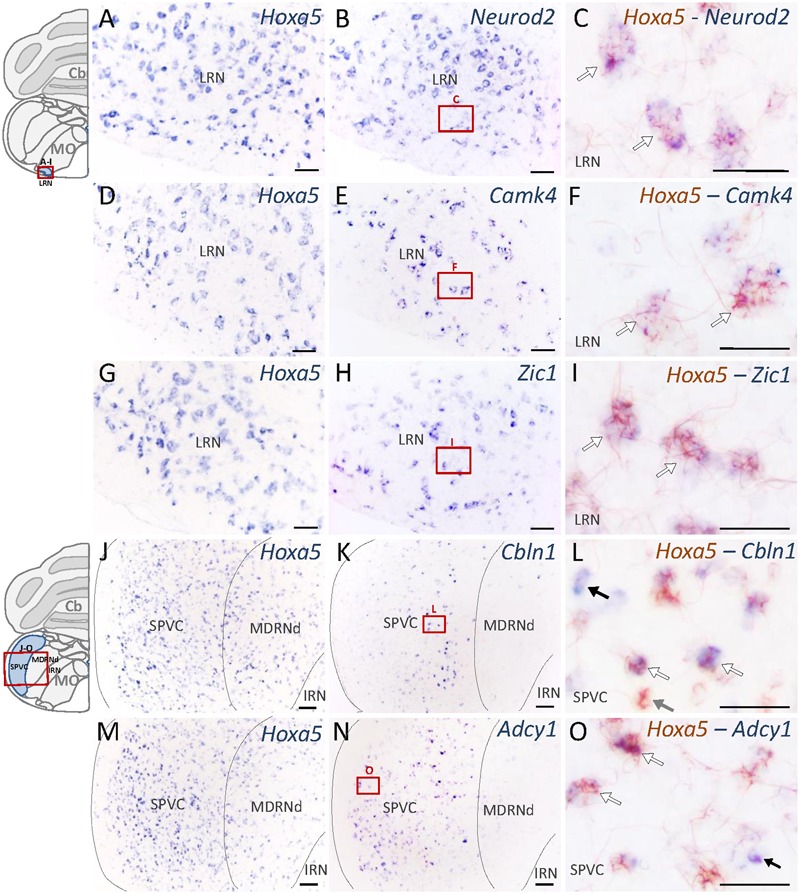
Expression of HOXA5 candidate target genes in the medulla oblongata analyzed by single and double *in situ* hybridization (ISH) on coronal cryosections of P21 mouse brains. Schematic views of coronal brain sections illustrate brain structures while square red boxes on the schemes refer to the localization of single ISH pictures. In double ISH (right column) *Hoxa5* signal is in red/brown precipitate while target gene signal is in blue. Localization of double ISH pictures is illustrated by square red boxes on the single ISH pictures (middle column). **(A–C)** Signal of *Hoxa5* and *Neurod2* in the lateral reticular nucleus (LRN). **(D–F)** Signal of *Hoxa5* and *Camk4* in LRN. **(G–I)** Signal of *Hoxa5* and *Zic1* in LRN. **(J–L)** Signal of *Hoxa5* and *Cbln1* in the caudal part of the spinal nucleus of the trigeminal (SPVC). **(M–O)** Signal of *Hoxa5* and *Adcy1* in SPVC. As illustrated in the right column, many *Hoxa5*-positive cells also show labeling for target gene transcripts. White arrows point to examples of co-localization, while gray arrows show example of cells only positive for *Hoxa5*, and black arrows show example of cells only positive for target genes. *n* = 2–4 brains hybridized for *Hoxa5* and each target genes in single and double ISH. Cb, cerebellum; MO, medulla oblongata. Medial is on the right, lateral is on the left. Scale bar 200 μm in **(A,B,D,E,G,H)**; 500 μm in **(J,K,M,N)**; 100 μm in **(C,F,I,L,O)**.

Nuclei that showed the highest enrichment in candidate target genes are the PG, the tegmental reticular nucleus (TRN), the lateral reticular nucleus (LRN) and the external cuneate nucleus (Parys et al., 2012), which are all major precerebellar nuclei providing inputs almost exclusively to the cerebellum. By forming mossy fibers that synapse onto the cerebellar granule cells, those nuclei are essential for coordinated motor activity, motor learning, and procedural memory (Rodriguez and Dymecki, 2000; Fu et al., 2013). Expression of *Camk4, Neurod2, Vglut1, Zic1*, and *Zic2* was observed in the PG and TRN, in a pattern overlapping with *Hoxa5* expression (**Figure 4** and **Table 2**). *Cbln1, Neurod2, Vglut1*, and *Zic1* transcripts were also detected in the interfascicular trigeminal nucleus (IF5), another *Hoxa5*-expressing major precerebellar pontine nucleus (**Figure 4** and **Table 2**). Co-labeling by double ISH revealed that all *Hoxa5*-positive cells were also positive for *Camk4, Zic1*, and *Zic2* in these nuclei (**Figures 4C,F,I,L**). In the medulla oblongata, *Camk4, Cbln1, NeuroD2, Vglut1, Zic1*, and *Zic2* expression was detected in the LRN as *Hoxa5* (**Figure 5** and **Table 2**). As observed in the pons, all the *Hoxa5*-positive cells in the caudal part of LRN were also *Camk4, NeuroD2, Zic1*, and *Zic2*-positive, while only a portion of *Hoxa5*-positive cells were *Cbln1*-positive (**Figures 5C,F,I** and data not shown). In the external cuneate nucleus, signal for *Cbln1, Grin2c, Vglut1, Zic1*, and *Zic2* was detected (**Table 2**). For *Zic1* and *Zic2*, the signal seemed to completely co-localize with *Hoxa5* expression, while only a portion of *Hoxa5*-positive cells were *Cbln1*-positive (data not shown). In addition to major precerebellar nuclei, transcripts of target genes were also detected in minor precerebellar nuclei, which have a less prominent projection to cerebellum, as illustrated in the caudal part of the spinal nucleus of the trigeminal (SPVC) for *Adcy1* and *Cbln1* (**Figures 5K,N** and **Table 2**). In this nucleus, co-localization of *Adcy1* and *Cbln1* with *Hoxa5* was partial (**Figures 5L,O**).

Altogether, the expression data revealed regionalized expression of several HOXA5 candidate targets in the brainstem of P21 wild-type mice, in patterns that highly overlapped with the *Hoxa5*-expression pattern. Double ISH showed cellular co-localization of *Hoxa5* and candidate targets transcripts in many precerebellar nuclei, although in PG, TRN, IF5, and LRN candidate targets displayed broader expression profiles than *Hoxa5* (data not shown). This supports a regulation of these candidate target genes by HOXA5 in the precerebellar system, regulation that could be either direct or indirect.

## Discussion

To tackle HOXA5 functions in the pons and medulla oblongata after birth, we analyzed the transcriptional programs downstream of HOXA5 in the brainstem. We found that HOXA5 regulates many genes associated to synaptic function. Indeed, GO analysis revealed that numerous transcripts downregulated in *Hoxa5* mutants are involved in glutamate synapse, GABAergic synapse and calcium signaling. Using RT-qPCR, we confirmed and extended these data, validating the robustness of the analysis and suggesting region-specific regulation of HOXA5 on target genes. By ISH, we also revealed the co-expression of *Hoxa5* with its targets, especially in nuclei of the precerebellar system.

### Functions of Genes with Downregulated Expression in *Hoxa5* Mutant Brainstem

Strikingly, very few candidate target genes are components or essential regulators of the Wnt/β-catenin, FGF, and TGF-β/Activin/BMP signaling pathways that have been identified as downstream pathways of several HOX proteins in embryonic development, neural differentiation and tumorigenesis (Chen et al., 2005; Schiedlmeier et al., 2007; Bami et al., 2011; Makki and Capecchi, 2011; Donaldson et al., 2012; Carroll and Capecchi, 2015; Duan et al., 2015; Hrycaj et al., 2015; Roux et al., 2015; Karmakar et al., 2017; Zhang et al., 2017). Moreover, except from *Pcp2* (see Supplementary Table S3, line 17), none of the already identified direct or indirect targets of HOXA5 was significantly downregulated in our analysis (e.g., *Ptn, Fslt1*) (Sanlioglu et al., 1998; Chen et al., 2005; Boucherat et al., 2013). Our data support a model in which HOX transcription factors regulate the expression of different target genes depending on the ontogenetic context. In the postnatal brainstem, HOXA5 seems to specifically participate in the regulation of synaptic function.

**FIGURE 6:**
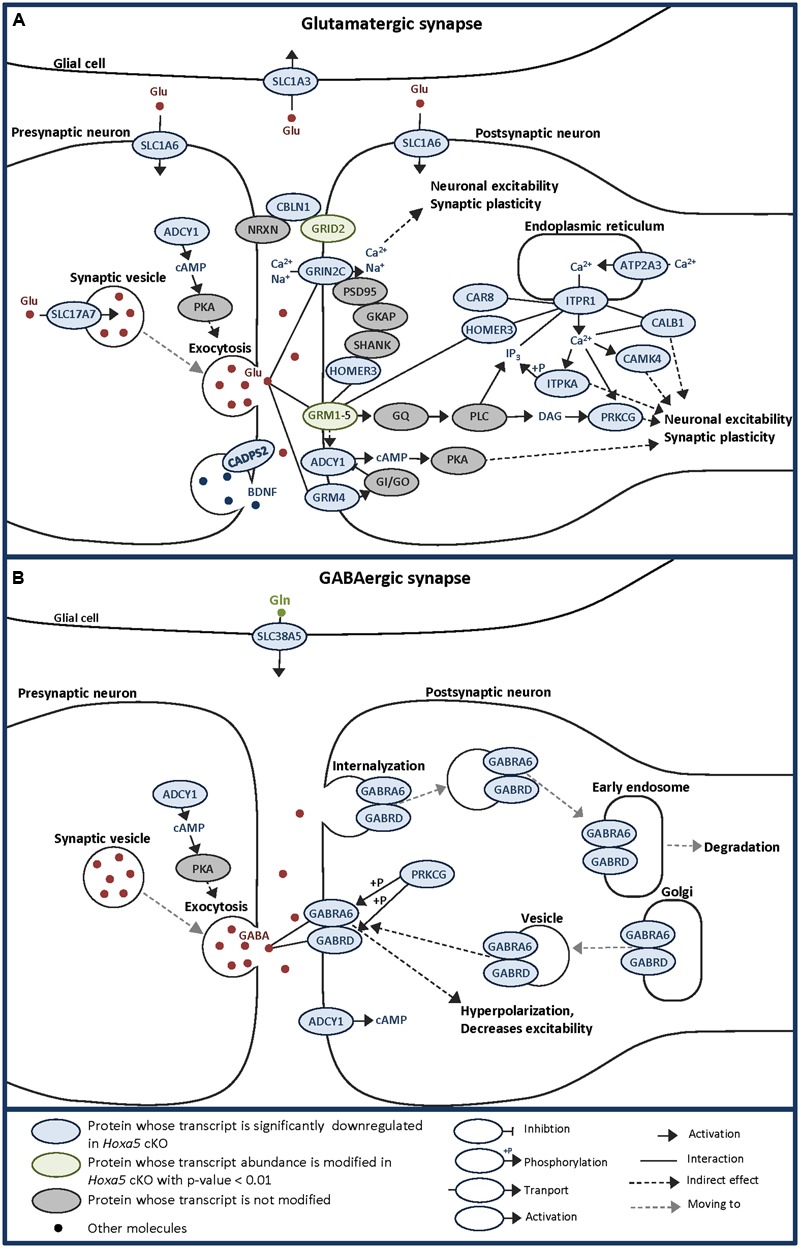
Schematic representations of glutamatergic **(A)** and GABAergic **(B)** synapses allowing to visualize the localization and potential links between HOXA5 potential targets identified by transcriptome analysis of P21 brainstem in this study. Adapted from KEGG pathway database. *Hoxa5* cKO: tamoxifen-treated *Hoxa5*^flox/flox^*;CMV-CreER*^T2+/-^; *Hoxa5* control: tamoxifen-treated *Hoxa5*^flox/flox^; *CMV-CreER*^T2-/-^.

Indeed, GO analysis and literature mining revealed that about 40% of the candidate genes are associated to the synapse, notably the glutamatergic synapse. A tentative synthesis of the functional links between proteins encoded by the HOXA5 candidate target genes associated to the glutamatergic synapse and calcium/calcium signaling is illustrated in **Figure 6A**, highlighting the importance of partnership between these molecules. Although the GABAergic synapse was not among the most significant enriched terms, the associated genes were among those whose fold change was the highest, such as *Gabra6* and *Gabrd* (**Figure 6B**).

Most of the downregulated transcripts in *Hoxa5* mutants involved in glutamatergic synapse are mainly postsynaptic while only a few are presynaptic (**Figure 6A**). Among the postsynaptic actors, we identified the two glutamate receptors: GRM4, a G protein-coupled receptor involved in the inhibition of cyclic AMP cascade, and GRIN2C, a NMDA receptor subunit that forms a channel permeable to calcium, potassium, or sodium depending on the other subunits. In the downstream pathway, we detected the calcium release mediator ITPR1, together with three of its interacting proteins, namely HOMER3, CAR8 and CALB1 (Tu et al., 1998; Hirota et al., 2003). Many proteins whose activity is influenced by calcium were identified, such as CAMK4, ADCY1, PRKCG, and ITPKA. We also detected the glutamatergic synapse organizer CBLN1, which interacts with NRXN and GRID2 to form a trimolecular trans-synaptic organizer (Hirai et al., 2005; Matsuda et al., 2010; Uemura et al., 2010; Ryu et al., 2012). Interestingly, GRID2 was also found downregulated in *Hoxa5* mutant specimens with a *p*-value <0.01. Through the regulation of these postsynaptic targets, HOXA5 could actively influence neuronal excitability and synaptic plasticity. Among the presynaptic actors of the glutamatergic synapse, we detected Slc17a7 (VGLUT1), which has an essential role during postnatal development by loading presynaptic vesicle with glutamate, thereby regulating the efficacy of neurotransmission (Wojcik et al., 2004). Another presynaptic target of HOXA5 is the Ca^2+^-dependent activator protein for secretion 2 (CADPS2), which enhances release of the neurotrophic factor BDNF involved in the functional maturation of the glutamatergic synapses and essential for normal postnatal cerebellar development (Gottmann et al., 2009; Sadakata and Furuichi, 2009). By regulating *Vglut1* and *Cadps2*, HOXA5 could thus control postnatal development of the cerebellum through the maturation of mossy fiber connections with the granular cells. This hypothesis is also supported by the downregulation of *Zic1* transcripts in *Hoxa5* cKO brainstem, which encode a Zinc-finger transcription factor involved in mossy fiber development (Dipietrantonio and Dymecki, 2009).

Although few members of GABAergic synapses are downregulated in *Hoxa5* mutants, they are specific actors of cerebellar function. Indeed *Gabra6* has been reported to have a very restricted expression in granule cells within the cerebellum and in a subset of precerebellar nuclei (Funfschilling and Reichardt, 2002; Maejima et al., 2013). This GABAa receptor subunit was shown to associate to the δ subunit encoded by *Gabrd* in the cerebellar granule cells, where they could impact on processes underlying motor coordination (Hanchar et al., 2005).

Together, these results support a new role for HOXA5 as a critical regulator of synaptic function, such as glutamatergic synapse integrity, excitability and/or plasticity, during the first three postnatal weeks.

### A Role for *Hoxa5* in the Development of Postnatal Cerebellar Pathways

A key feature of *Hoxa5* expression in the postnatal and adult hindbrain is its enrichment in precerebellar nuclei in the pons and medulla oblongata (Lizen et al., 2017). Neurons of the precerebellar system, which relay both peripheral sensation and cortical input to the cerebellum, derive from the caudal part of the rhombic lip (r6–r8), using tangential migration to reach their final position. It has been suggested that a rhombomere-specific HOX code controls appropriate precerebellar neuron position during tangential migration as well as appropriate cortical input on pontine nuclei. Notably, PG5 HOX proteins were shown to be functionally implicated in the topographic organization of migrating precerebellar pontine neurons (Di Meglio et al., 2013).

Strikingly, a high proportion of significantly downregulated genes identified in the brainstem of *Hoxa5* cKO mice at P21 are associated to the cerebellum. The ISH analysis revealed that these candidate target genes are also expressed in the precerebellar nuclei, where several co-localize at the cellular level with *Hoxa5*. These results suggest a postnatal requirement of HOXA5 in these nuclei for processes beyond the neuronal migration, such as activity-dependent refinement of neuronal pre-established circuits. As cerebellum grows and matures essentially during the postnatal period, cerebellar and precerebellar circuits are particular vulnerable to environmental signals (Wang and Zoghbi, 2001), as shown for pontine neurons that carry on their maturation during the first weeks after birth. Indeed, around P1, pyramidal tract fibers from the cortex grow toward the PG where they enter and establish synapses. Refinement of cortico-pontine projections is then observed by the decreasing of collateral branches around P16 (Inoue et al., 1991; Leergaard et al., 1995). During this postnatal refinement, both presynaptic (cortico-pontine axons) and postsynaptic (pontine neurons) elements play an active role. Our data support a role for HOXA5 in cortico-pontine projections refinement through regulation of transcripts involved in postsynaptic ending on pontine neurons. Of note, calcium and CAMK4 are part of signaling mechanisms involved in the regulation of dendrite growth, branching, and elongation (Nagendran and Hardy, 2011). A role for ADCY1 in the regulation of synapse stabilization/elimination during the late stage of fine topography of sensory circuits has also been demonstrated (Nicol and Gaspar, 2014). Finally, through its regulation of *Wnt7a*, HOXA5 could influence synapse development, remodeling, and maintenance at both presynaptic and postsynaptic levels (Dickins and Salinas, 2013). This hypothesis is further supported by the involvement of HOX factors in refinement of connectivity in brainstem cochlear neurons (Karmakar et al., 2017), where PG2 HOX proteins present in the postsynaptic neurons, the glutamatergic bushy cells, orchestrate the tonotopic precision of pre-synaptic input.

HOXA5 could also influence the postnatal maturation of pontine output in the granular layer of the cerebellum through regulating presynaptic actors such as VGLUT1 and CADPS2 mentioned above. Involvement of HOXA5 in the postnatal maturation of cortico-ponto-cerebellar connections could affect the different functions in which these circuits are involved such as procedural memory, motor learning, and motor coordination. In this context, it is worth mentioning that our GO analysis revealed enrichment in associated terms (e.g., long-term memory and long-term potentiation).

As PG2 to PG5 *Hox* genes are expressed in pontine nuclei from fetal to postnatal stages (Hutlet et al., 2016), they may share different functions in postnatal maturation of cortico-ponto-cerebellar circuits. Expression of candidate target genes in anterior part of pontine nuclei, where *Hoxa5* is not expressed, suggests co-regulation by HOX proteins expressed more anteriorly in pontine nuclei. These proteins could regionally specify cortico-ponto-cerebellar projections during postnatal maturation through regulation of specific transcriptional codes in each pontine nuclei sub-population. As such, *Hox* genes could be involved in postnatal activity-dependent refinement of topographic precision of connectivity. In support of this hypothesis, a few of our candidate targets genes, such as *Cadps2, Camk4, NeuroD1, Zic1, Cbln1*, were identified downstream of other HOX proteins (Bami et al., 2011; Makki and Capecchi, 2011; Anderson et al., 2013; Karmakar et al., 2017).

### HOXA5 in Synaptic Function and Dysfunction

Glutamatergic synapse dysfunction has profound effects both in neurological diseases and psychiatric disorders such as Parkinson and Alzheimer diseases, depressive disorders, schizophrenia, and autism spectrum disorder (Ravasi et al., 2010). Dysfunction of these synapses in the first week postnatally could impact on ASD. In this context, several genes downregulated in the brainstem of *Hoxa5* cKO mouse at P21, such as *Grm4, Cbln1, En2, Camk4*, and *Cadps2*, have been associated to autism traits either in humans or in mouse models (Sadakata and Furuichi, 2009; Becker et al., 2014; Waltes et al., 2014; Genestine et al., 2015; Krishnan et al., 2017). Moreover, several HOXA5 candidate target genes identified such as *Cbln1, Cbln3, Homer3, Grm4, En2, Atp2a3, Fat2*, and *Car8*, are also downregulated in a recent mouse model of ASD, generated by increasing dosage of E3 ubiquitin ligase UBE3A, which recapitulates a highly penetrant type of autism in humans (Smith et al., 2011; Krishnan et al., 2017). Although HOXA5 protein has not been detected in the forebrain-derived territories, which have been mostly investigated in deciphering ASD underlying pathways, recent analysis highlights a role for the cerebellum in the perinatal risks for autism (Wang et al., 2014). Therefore, these data point to HOXA5 as a potential regulator in pathways that are affected in ASD.

## Conclusion

Our molecular phenotyping of *Hoxa5* cKO brainstem at P21 highlights a downregulation of many genes associated with synaptic function in *Hoxa5* mutant. GO analysis and literature mining allowed us to point key actors involved in glutamatergic synapse, calcium signaling pathway, and GABAergic synapse. In agreement with our previous hypothesis, this study supports a new role for HOXA5 in the establishment and refinement/plasticity of precerebellar circuits after birth. Indeed, HOXA5 could orchestrate the cortico-ponto-cerebellar circuit formation and maturation during the postnatal period. As such it could modulate important cerebellar functions such as motor coordination, motor learning, and procedural memory, and its alteration could impact on the development of synaptopathies such as ASD.

## Author Contributions

BL and FG designed the research. LJ provided the mouse transgenic line. BL, CM, JM, TS, M-TA, and AS performed the research. BL, CM, JM, AS, and FG analyzed the data. BL and FG wrote the article with all other authors and LJ revised it critically.

## Conflict of Interest Statement

The authors declare that the research was conducted in the absence of any commercial or financial relationships that could be construed as a potential conflict of interest.

## Footnotes

1 http://ccb.jhu.edu/software/tophat/index.shtml

2 http://cole-trapnell-lab.github.io/cufflinks/manual/

3 https://www.ncbi.nlm.nih.gov/geo/query/acc.cgi?acc=GSE103390

4 https://www.ncbi.nlm.nih.gov/pubmed

## Supplementary Material

TABLE S2 Primers and parameters of quantitative real-time RT-qPCR analysis.
