# Supplementary material for: Conditional Loss of Hoxa5 Function Early after Birth Impacts on Expression of Genes with Synaptic Function
Source: Front Mol Neurosci. 2017 Nov 15;10:369. doi: 10.3389/fnmol.2017.00369 (PMC5695161; doi:10.3389/fnmol.2017.00369)
Supplement: Supplementary file 1 [file Table_1.DOCX]

| PCR | Step | Temp°C | Time | Note | Primer type | Primers Sequence 5’ 🡪 3’ |
| --- | --- | --- | --- | --- | --- | --- |
| *Hoxa5^flox^* | 1 | 94 | 3 min |  | Hoxa5 loxp Forward | TCT GAT TTA TGC CAA ATA GCA AGC T |
|  | 2 | 94 | 30 sec |  | Hoxa5 loxp Reverse | GAA AGA CGG CAT CCG TGT AAG |
|  | 3 | 58 | 30 sec |  |  |  |
|  | 4 | 72 | 1 min | repeat steps 2-4 for 40 cycles |  |  |
|  | 5 | 72 | 10 sec |  |  |  |
|  | 6 | 4 | - | hold |  |  |
| *CMV-CreER^T2^* | 1 | 95 | 5 min |  | Cre Forward | GTC CGG GCT GCC ACG ACC AA |
|  | 2 | 94 | 1 min |  | Cre Reverse | ACG GAA ATC CAT GCG TCG ACC AGT T |
|  | 3 | 65 | 1 min |  |  |  |
|  | 4 | 72 | 30 sec | repeat steps 2-4 for 33 cycles |  |  |
|  | 5 | 72 | 10min |  |  |  |
|  | 6 | 4 | - | hold |  |  |

**Table S1:** PCR program and primers sequences
