# Supplementary material for: Conditional Loss of Hoxa5 Function Early after Birth Impacts on Expression of Genes with Synaptic Function
Source: Front Mol Neurosci. 2017 Nov 15;10:369. doi: 10.3389/fnmol.2017.00369 (PMC5695161; doi:10.3389/fnmol.2017.00369)
Supplement: Supplementary file 6 [file Image_1.PDF]

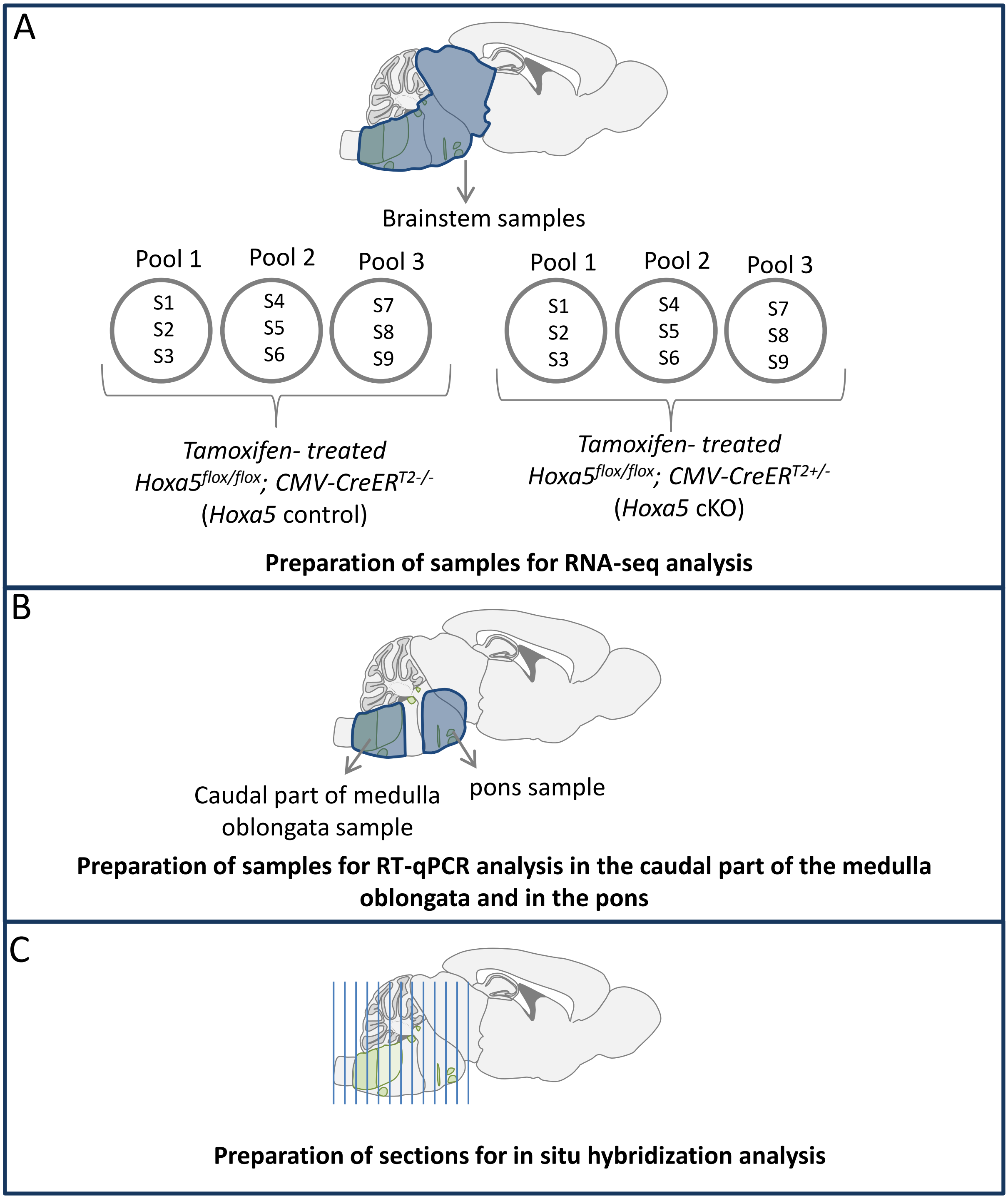

Figure S1. Experimental flowchart for characterization of *Hoxa5* downstream target genes in the postnatal mouse brain.

(A) For RNA-seq analysis, brainstem samples (S) were dissected from tamoxifen-treated $Hoxa5^{flox/flox}$; $CMV\text{-}CreER^{T2-/-}$ (*Hoxa5* control) and $Hoxa5^{flox/flox}$; $CMV\text{-}CreER^{T2+/-}$ (*Hoxa5* cKO) P21 mice. A total of 9 mice were used per genotype. For each genotype, three brainstem samples were pooled, and three pools were generated. (B) For RT-qPCR analysis, P21 brainstem samples from *Hoxa5* control and *Hoxa5* cKO mice were dissected to isolate the caudal part of the medulla oblongata and the pons. This procedure aims to improve the accuracy of the analysis of differentially expressed genes between the two genotypes. (C) The neuroanatomical pattern of *Hoxa5* and candidate target genes was analyzed by in situ hybridization on 14µm coronal cryosections of P21 brainstem from wild type mice.
